# Cost effectiveness of community led total sanitation in Ethiopia and Ghana

**DOI:** 10.1016/j.ijheh.2020.113682

**Published:** 2021-03

**Authors:** Jonny Crocker, David Fuente, Jamie Bartram

**Affiliations:** a Department of Global Health, University of Washington, 325 9th Avenue (Box 359931), Seattle, WA, 98104, USA; b School of Earth, Ocean and Environment, University of South Carolina, Columbia, SC, USA; c Department of Environmental Sciences and Engineering, University of North Carolina at Chapel Hill, USA; d University of Leeds, England

**Keywords:** CLTS, Sanitation, Open defecation, Cost efficiency, Cost effectiveness, Economic

## Abstract

We conducted cost effectiveness analyses of four different CLTS interventions implemented in Ethiopia and Ghana. In each country, a pilot approach in which additional local actors were trained in CLTS facilitation was compared to the conventional approach. Data were collected using bottom-up costing, household surveys, and observations. We assessed variability of cost effectiveness from a societal perspective for latrine ownership and latrine use outcomes in different contexts. Cost effectiveness ranged from $34–$1897 per household ($5.85–$563 per person) gaining access to a private latrine or stopping open defecation, depending on the intervention, context, and outcome considered. For three out of four interventions, CLTS appeared more cost effective at reducing open defecation than at increasing latrine ownership, although sensitivity analysis revealed considerable variation. The pilot approaches were more cost effective at reducing open defecation than conventional approaches in Ethiopia, but not in Ghana. CLTS has been promoted as a low-cost means of improving the ownership and use of sanitation facilities. In our study, the cost of CLTS per household gaining latrine access was slightly higher than in other studies, and the cost of CLTS per household stopping OD was slightly lower than in other studies. Our results show that aggregate measures mask considerable variability in costs and outcomes, and thus the importance of considering and reporting context and uncertainty in economic analysis of sanitation interventions.

## 1 Introduction

In 2010, the United Nations recognized sanitation as a human right (United Nations General Assembly, 2010). The Sustainable Development Goals (SDGs) include behavioral and infrastructure targets for sanitation (UN General Assembly, 2015), that require 892 million people switching from open defecation (OD) to latrine use, and 2.3 billion people gaining access to a private latrine (WHO/UNICEF, 2017). Open defecation and lack of safe sanitation are associated with increased diarrhea and mortality, and negative social impacts among women and children (Bisung and Elliott, 2017; Prüss-Ustün et al., 2019; Wolf et al., 2018).

Interventions for changing sanitation behavior and increasing access to private latrines span from no-subsidy demand generation to latrine provision to microfinance. Economic analysis can be used to identify economically attractive development programs (Hutton et al., 2014; Trémolet et al., 2010; Whittington et al., 2012). Community-led total sanitation (CLTS) is an approach for generating demand for sanitation that has quickly reached wide-scale implementation in over 50 countries since it was introduced in 2000. The rapid expansion of CLTS preceded evidence generation, and was driven largely by perceptions that it was cheap and fast (Attanasio et al., 2004). Evidence on the effectiveness of CLTS is growing, with many recent published evaluations (USAID, 2018; Venkataramanan et al., 2018). These show that CLTS can reduce OD and increase access to private latrines (Pattanayak et al., 2009; Pickering et al., 2015); however, it can also be ineffective (Guiteras et al., 2015). Evidence on the cost and cost effectiveness of CLTS has not kept pace with evidence on effectiveness. There are only a few prior studies that report economic outcomes for CLTS or a related intervention (Briceño and Chase, 2015; Trémolet et al., 2010; Woode et al., 2018). A recent study modeling benefit-cost ratios for CLTS relied mostly on assumptions for estimating CLTS costs, due to the lack of empirical data available (Radin et al., 2019). They found that CLTS can be cost-beneficial in many but not all situations.

Evaluations of sanitation interventions tend to report aggregate effect estimates, even for multi-site studies. The impact of sanitation interventions on latrine coverage, use, and health outcomes varies dramatically between studies, and the setting in which interventions occur can be a determinant of the outcomes (Garn et al., 2017; Schmidt, 2015). There have been several global economic analyses in the water, sanitation, and hygiene (WaSH) sector (Hutton et al., 2007). However, local context conditions the cost and outcomes of WaSH programs (Whittington, 2015). Thus, these global averages do not provide useful information about the relative performance of sanitation investments in specific contexts. Ideally, sanitation economic evaluations should study variation between sites, settings, and intervention delivery mechanisms (Kremer and Zwane, 2007). This would yield a more nuanced sense of where programs are likely to succeed or meet particular investment criteria.

This paper presents the results of a retrospective (ex-post) cost effectiveness analysis to highlight the importance of incorporating local context and uncertainty in economic analysis. We report the cost effectiveness of four different CLTS interventions in five regions in Ethiopia and Ghana. This study had two research questions: 1) what is the cost effectiveness of CLTS in reducing OD and increasing latrine ownership, and 2) to what extent does cost effectiveness vary between contexts and with uncertainty in key parameters. We examine costs from a societal perspective, report cost effectiveness for two different outcome measures (reductions in OD and increases in latrine ownership), and report how cost effectiveness varies between different regions. This study follows the CHEERS reporting guideline (Husereau et al., 2013).

## 2 Methods

### 2.1 Program description

Four different CLTS interventions were implemented: in Ethiopia, (1) health extension worker and kebele leader-facilitated CLTS (“HEW CLTS”), and (2) teacher-facilitated CLTS (“Teacher CLTS”); and in Ghana, (3) NGO-facilitated CLTS (“NGO CLTS”), and (4) NGO-facilitated CLTS with additional training for natural leaders (“CLTS + NL Training”) (Table 1). A kebele is the lowest administrative unit in Ethiopia, comprising 20–30 villages and approximately 5000 people in rural areas. Natural leaders (NLs) are motivated community members who encourage others to construct latrines and change sanitation-related behaviors. Facilitation comprised visits to study villages by facilitators to conduct the three typical stages of CLTS: pre-triggering (community entry), triggering, and follow-up, which involves monitoring a community's progress and guiding them toward eliminating OD.

**Table 1:** Differences between interventions analyzed.

Intervention	Facilitation
HEW CLTS (Ethiopia)	Health extension workers and kebele leaders
Teacher CLTS (Ethiopia)	Teachers
NGO CLTS (Ghana)	NGO staff
CLTS + NL Training (Ghana)	NGO staff and natural leaders

This table describes the main distinguishing factor between each intervention. Other differences are more thoroughly described in a previous publication (Crocker et al., 2017b).

The two interventions in Ethiopia lasted 12 months, and the two in Ghana lasted 18 months. The interventions in Ethiopia took place in the Oromia and Southern Nations, Nationalities, and Peoples (SNNP) regions, and in Ghana in the Central, Upper West, and Volta regions. Interventions 1 and 2 in Ethiopia began with Plan International (Plan) training local actors who then led CLTS facilitation. Interventions 3 and 4 in Ghana were facilitated by Plan. Plan's interest in this study was to evaluate if pilot interventions (interventions 2 and 4) would improve the cost effectiveness of the conventional CLTS interventions in Ethiopia and Ghana, which were facing challenges as CLTS implementation was scaled up. There were several pre-existing enabling factors, including supportive national governments, local governments tasked with implementing CLTS, and a strong partnership between Plan and UNC (Saywell and Crocker, 2019). Further program details are available in implementation narratives and situational assessments on the project website (waterinstitute.unc.edu/clts).

### 2.2 Study design

Study design, data collection, rationale for village selection, and costing methods are described in previous publications (Crocker et al, 2016a, 2016b, 2017a, 2017b). Briefly, the study in Ethiopia used a quasi-experimental design, in which six kebeles (165 villages) were matched on latrine access and population, then manually assigned to receive CLTS facilitated by either HEWs and kebele leaders, or by teachers. The study in Ghana used a cluster-randomized design, in which all 58 project villages received CLTS, and 29 of the villages were randomly selected to receive NL training as an add-on activity. A survey of a representative sample of households (2182 in Ethiopia, 1594 in Ghana) was used to measure sanitation outcomes and household spending on latrines. Surveys in Ethiopia were administered immediately before implementation began, and again 2 years later (1-year after implementation ended). Surveys in Ghana were administered 2.5 years after implementation began (1-year after implementation ended). In Ghana, household recall on latrine age was used to estimate baseline coverage. Surveys covered demographics and WaSH indicators, and included latrine observations. All data collection was conducted in local languages by an experienced independent contractor. Printed surveys were used in Ethiopia, and SurveyCTO software on Nexus tablets was used in Ghana.

Costs were assessed using a bottom-up, activity based costing method. Costs were measured (rather than estimated). Plan used checklists to track their implementation activities in detail (including management, training, and facilitation). Financial expenditures and receipts were reviewed to generate unit costs (e.g. for training venue rental, transportation, and person-time). Household surveys were used to collect data on community members' time and money spent on sanitation during the study period. Costs were categorized as program cost (management, training, facilitation), and local costs (local actor time, community member time, and latrine spending). The management category includes time spent planning and preparing for the interventions. Training and facilitation include transportation costs. Local actors’ unpaid time was monetized using value-of-time estimates generated through review of local wages and survey results. No discount rate was used as costs were evaluated over a 2-year period (2012–2014). Ethical clearance was obtained from the UNC Institutional Review Board, and from the appropriate institutions in Ethiopia and Ghana. Informed consent was obtained from all study participants.

### 2.3 Analysis

Cost effectiveness of the CLTS interventions was calculated using two outcome measures (reduced OD, and increased latrine ownership). Latrine use included use of communal, shared, and private latrines. Self-reported private latrine use was validated by observing latrines. Households who reported using latrines that were full, unstable, or could not be observed were categorized as OD. Similarly, households were categorized as owning a latrine only if it had stable flooring and was not full on the day of surveying. The difference between self-reported behaviors and observation of a usable latrine are reported in a prior publication (Crocker et al., 2017a). Others have also pointed out that self-reported latrine use is an imperfect proxy for latrine use, although it is used for measuring the SDG indicators (Thomas et al., 2018).

The assessment of cost effectiveness of the CLTS interventions takes a societal perspective by including both program and local costs (Fuente et al., 2012). Program costs were those borne by Plan, which included management, training, and facilitation. Program costs were assessed through implementation tracking of Plan's CLTS interventions, and review of financial expenditures. Local costs were those borne by local actors (district government, teachers, health workers, community members), comprising the economic value of their time, and households' financial expenditures on latrines. Local costs were assessed through a combination of Plan's implementation tracking, and through household and local actor surveys.

To arrive at cost effectiveness, program and local costs were divided by the number of households stopping OD or gaining latrine ownership. Cost effectiveness values should be interpreted as the societal cost to convert a household to latrine use or latrine ownership.

CE_i_ = Cost / Outcome_i_

Where i = OD or latrine ownership. Sensitivity of cost effectiveness to uncertainty about the costs and outcomes of the programs was assessed using Monte Carlo analysis, using assumptions summarized in the supplement. Costs were measured, not estimated, so a uniform distribution with cost range of ± 30% from base values presented in Table 1 was chosen as a conservative approach for sensitivity analysis. For outcomes, normal distributions using standard deviations taken from impact evaluation data were used. Standard deviations for changes in open defecation in Ghana were not available, so 50% of the point estimates were used for the Monte Carlo analysis as a conservative approach, which is a larger range than used in previous studies (Radin et al., 2019; Whittington et al., 2012). Monte Carlo analysis was implemented using Oracle Crystal Ball and 1000 draws of the parameter values.

## 3 Results

### 3.1 Study population

Large differences existed between the Ethiopia and Ghana study populations. The Ethiopia study population had more people per household, indications of lower wealth, smaller villages, lower access to an improved water supply, and higher latrine ownership than the Ghana study population. Within both countries, there were regional differences; the Oromia region in Ethiopia and the Upper West region in Ghana showed larger households, and lower wealth, access to improved water supply, and latrine ownership (Table 2). A breakdown of these variables by intervention is in the appendix.

**Table 2:** Household and respondent characteristics in villages receiving CLTS in Ethiopia and Ghana, by region.

Variable	Ethiopia			Ghana			
	All regions	Oromia	SNNP	All regions	Central	Upper West	Volta
Female respondent	76%	74%	77%	71%	68%	95%	62%
Five or more years of education	18%	13%	21%	42%	57%	15%	75%
Household size	5.8	6.1	5.6	4.9	3.3	6.4	3.6
Number of children per household	0.9	1.0	0.9	0.6	0.5	1.1	0.5
Metal roof	21%	11%	29%	91%	97%	72%	94%
Own radio	26%	30%	24%	49%	43%	49%	55%
Own television	1%	0%	1%	37%	46%	19%	38%
Years family lived in village	22	25	21	28	27	36	25
Years family lived in current house	14	6	18	14	14	16	15
Access to improved water supply	21%	4%	33%	59%	65%	41%	60%
Baseline latrine ownership	78%	51%	98%	21%	24%	10%	25%
Baseline open defecation	45%	70%	27%	49%^c^	34%^c^	96%^c^	36%^c^
Village size	34	29	38	116	164	68	123
Villages with prior WaSH projects	0%	0%	0%	72%	100%	45%	79%
Villages with prior subsidized latrines	0%	0%	0%	28%	33%	15%	37%

All Ghana values are taken from the 1.5-year follow up household census and survey, and describe the two treatment groups at that time, except for baseline private latrine ownership, which is based on recall of how old their latrines were. The education variable assumes that respondents who have completed primary education in Ghana have spent at least five years in education. Baseline surveys were not used in Ghana, so baseline open defecation was based on the conservative assumption that decreases in open defecation were equivalent to increases in latrine ownership. “Access” meaning the improved water supply is within 30 min walking distance.

### 3.2 Cost effectiveness

Table 3 presents cost and outcome data and cost effectiveness for the four CLTS interventions by region for two outcome measures. For the full study population across both Ethiopia and Ghana in aggregate, CLTS cost $358.87 per household stopping open defecation, and $530.63 per household gaining ownership of a usable latrine. This equates to $75.27 *per person* stopping open defecation, and $111.29 *per person* gaining ownership of a usable latrine (data not shown).

**Table 3:** Cost effectiveness of four CLTS interventions in Ethiopia and Ghana.

	Variable	Both countries all regions	Combined Ethiopia regions		Oromia region		SNNP region		Combined Ghana regions		Central region		Upper West region		Volta region		
		All CLTS interventions	CLTS	Teacher-CLTS	CLTS	Teacher-CLTS	CLTS	Teacher-CLTS	CLTS	CLTS + natural leader training	CLTS	CLTS + natural leader training	CLTS	CLTS + natural leader training		CLTS	CLTS + natural leader training
	**Column ID**	**A**	**B**	**C**	**D**	**E**	**F**	**G**	**H**	**I**	**J**	**K**	**L**	**M**		**N**	**O**
	Households	12,217	1624	3838	651	1586	973	2252	3443	3312	1463	1495	808	540		1172	1277
	People	58,248	9829	21,216	4013	9153	5816	12,063	14,263	12,940	4670	5041	5208	3474		4385	4425
Outcomes	Change in open defecation	−13%	−10%	−11%	−48%	−56%	15%	21%	−9%	−22%	−6%	−16%	−13%	−53%		−9%	−15%
	Change in usable latrine ownership	9%	4%	6%	43%	53%	−22%	−27%	9%	15%	7%	4%	8%	33%		12%	19%
	Households stopping open defecation	1605	168	421	310	895	−141	−474	298	718	89	237	108	287		100	195
	Households gaining ownership of a usable latrine	1086	70	229	282	841	−211	−612	305	481	102	58	634	179		139	243
Costs	Management cost	$79,660	$8690	$14,867	$4345	$7434	$4345	$7434	$26,958	$29,145	$8797	$9525	$9308	$10,037		$8853	$9582
	Training cost	$210,850	$17,617	$33,229	$9979	$17,279	$7638	$15,951	$4076	$155,928	$1199	$38,427	$928	$62,874		$1949	$54,627
	Facilitation cost	$169,595	$4891	$6225	$2820	$1118	$2071	$5107	$73,428	$85,052	$28,573	$32,281	$18,270	$23,209		$26,584	$29,562
	Local actor time cost	$12,583	$1926	$3863	$1084	$1889	$842	$1974	$1242	$5552	$314	$1525	$372	$1998		$555	$2030
	Community time cost	$21,529	$2546	$4151	$1435	$1886	$1110	$2265	$5847	$8985	$1502	$3661	$1452	$2028		$2893	$3297
	Hired labor cost	$21,942	$0	$0	$0	$0	$0	$0	$7132	$14,810	$1096	$1835	$2191	$3859		$3845	$9115
	Hardware cost	$59,901	$1070	$1013	$911	$633	$159	$380	$13,101	$44,718	$4319	$23,501	$1282	$2452		$7501	$18,765
	Total cost	$576,061	$36,739	$63,348	$20,573	$30,239	$16,166	$33,110	$131,783	$344,190	$45,800	$110,754	$33,803	$106,458		$52,180	$126,978
**Cost effectiveness**	**Cost per household stopping open defecation**	**$358.87**	$218.26	$150.65	$66.44	$33.79	*NA*		$442.26	$479.11	$512.01	$467.50	$311.86	$371.43		$521.12	$651.62
	**Cost per household gaining usable latrine ownership**	**$530.63**	$522.02	$276.53	$73.03	$35.95			$432.11	$715.29	$447.24	$1897.01	$531.31	$593.22		$375.54	$521.80

Sanitation outcomes worsened in the SNNP region, yielding misleading negative cost effectiveness ratios, so they are not presented in this table. Changes in open defecation and latrine ownership are for a 2.5-year period for Ghana, and a 2-year period for Ethiopia (baseline to 1-year after the interventions ended). Cost effectiveness estimates assume open defecation would not have changed in the absence of the CLTS interventions. Management, training, and facilitation costs were borne by Plan. Local actor and community time are economic costs. Hired labor and hardware costs were borne by households. “Usable” latrines were not full and had stable flooring on the day of surveying. Changes in open defecation and latrine ownership in Ghana are based on recall of latrine age. A previous publication explores variation in cost within and between countries(Crocker et al., 2017b)

In Ethiopia, the pilot intervention was more cost effective than conventional CLTS for both outcomes (Table 3, comparing column B to C). However, in Ghana, the pilot intervention was *less* cost effective than conventional CLTS for both outcomes (comparing column H to I). Both of the CLTS interventions in Ethiopia were more cost effective than those in Ghana (comparing columns B and C to H and I). For example, the two interventions in Ethiopia cost $218.26 and $150.65 per household stopping OD, while the two interventions in Ghana cost $442.26 and $479.11 per household stopping OD. In most cases, it was cheaper to reduce OD than it was to increase latrine ownership, with conventional CLTS in the Central and Volta regions being the exceptions (comparing the last two rows). This was due to some of the reduction in OD coming from latrine sharing, as discussed in previous publications (Crocker et al., 2016a, 2016b). Incremental cost effectiveness ratios are presented in Table A3 in the supplement.

In Ethiopia, both CLTS interventions were more cost effective in the Oromia region. In the SNNP region, open defecation and latrine ownership did not improve, which would yield negative cost effectiveness ratios so they are not presented in Table 3. In Ghana, the CLTS interventions were more cost effective at reducing OD in the Upper West region, and more cost effective at increasing latrine ownership in the Volta region where latrine ownership increased more than latrine use (columns L, M, N, O).

**Fig. 1:**
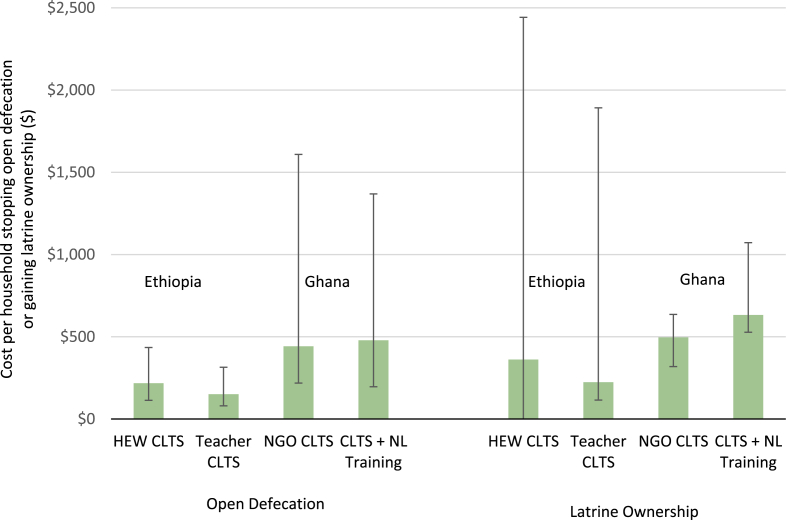
Cost effectiveness of four different CLTS interventions per household for two outcome measures (stopping open defecation or gaining ownership of a private latrine) in Ethiopia and Ghana. Error bars represent 95% confidence intervals from Monte Carlo simulations. Lower-bound confidence interval for latrine ownership results in Ethiopia are truncated at zero.

Fig. 1 summarizes the Monte Carlo sensitivity analysis for cost effectiveness of the CLTS interventions in Ghana and Ethiopia for two outcome measures (OD and latrine ownership). Cost effectiveness was sensitive to uncertainty about the costs and outcomes of the relative interventions. For example, the 95% confidence intervals from the Monte Carlo simulations do not indicate that one intervention was more cost effective than the other for either of the outcome measures.

## 4 Discussion

Since its first introduction in Bangladesh in 1999, CLTS has been promoted as a low cost means of improving the ownership and use of improved sanitation facilities (Kar and Chambers, 2008). In our study, the cost of CLTS per household gaining latrine access was $530.63 in aggregate, which is slightly higher than related interventions. An evaluation of a World-Bank funded program in Tanzania reported the cost of gaining access to a latrine as $194 per household for a sanitation-promotion program, and $491 for a sanitation and hygiene promotion program (Briceño and Chase, 2015). Another study reported a ratio of $344 per household gaining latrine access in Senegal (Trémolet et al., 2010). Our study found the cost of CLTS per household stopping OD was $358.87 in aggregate. We are not aware of previous studies reporting the cost per household stopping OD for a CLTS intervention. A study of a small hygiene promotion program in four villages in Ghana saw costs per household changing any hygiene behavior (latrine use, handwashing, or safe water use) of $532, which is higher than the cost per household stopping OD in our study (Woode et al., 2018). They used improvement in any of three WaSH behaviors as their outcome, making direct comparison less conclusive. Care should be taken when interpreting these direct comparisons between studies, due to methodological differences. For example, two of the three cited studies used top-down costing, which may result in underestimated costs and lower cost effectiveness ratios.

The pilot CLTS interventions yielded improved sanitation outcomes over conventional CLTS in both Ethiopia and Ghana—a greater portion of households within project communities stopped open defecation and gained ownership of a usable private latrine. Additionally, pilot CLTS was more cost effective in aggregate than the conventional interventions at reducing OD and increasing latrine ownership in Ethiopia. For the full sample (all regions and interventions; Table 3, column A), OD reduced by 13% while latrine use increased by 9%, indicating that CLTS was 44% more cost effective at reducing OD than increasing latrine use. This may indicate that for CLTS, moving households up the sanitation ladder (WHO/UNICEF, 2017) is cheaper for the lowest rung on the ladder (stopping OD) than for higher rungs that require owning a private latrine.

Nevertheless, there was considerable variability in the cost effectiveness of the interventions, depending on intervention, country, region, and outcome measure. For example, in Ethiopia neither intervention improved sanitation outcomes in the SNNP region. This yields an aggregate cost effectiveness in Ethiopia of $151–$218 per household stopping OD, compared to a much more attractive cost effectiveness of $34–$66 per household stopping OD when considering the Oromia region alone. NL training in Ghana was more cost effective than conventional CLTS in the Central region using OD as an outcome measure. NL training was less cost effective than conventional CLTS in the Upper West and Volta regions. In an ex-ante cost effectiveness analysis, these results could be interpreted as a justification for implementing the NL intervention in the Central region and the conventional CLTS intervention in the Upper West and Volta regions. However, both latrine ownership and use outcomes were substantially (4X) higher with the inclusion of NL training in the Upper West region. CLTS outcomes have varied with implementation differences elsewhere as well (Harter et al., 2019). The higher spending in more intensive CLTS programs that include training more people within villages can improve latrine ownership and use within those villages and likely justify the costs, especially given emerging evidence that high neighborhood-level sanitation coverage is important for health impacts (Fuller and Eisenberg, 2016; Harris et al., 2017).

Cost effectiveness differed between Ethiopia and Ghana. This was largely driven by differences in costs, not in outcomes. It is also important to note that the quality of latrines resulting from CLTS interventions tended to be higher in Ghana (Crocker et al., 2017a). We do not think our results would justify prioritizing investing in CLTS in one country over another. We instead compare cost effectiveness between Ethiopia and Ghana to discourage others from directly extrapolating cost or cost effectiveness results between countries. We are not aware of any prior studies that report empirical economic data from the same sanitation interventions in multiple countries.

Cost effectiveness versus just effectiveness provide contrasting results in Ghana. The conventional CLTS intervention was more cost effectiveness, in aggregate and in each region, than the NL intervention in Ghana. However, the NL training had a greater impact on latrine ownership and OD in aggregate and in the Upper West and Volta regions than conventional CLTS, but that increase in outcomes came at a cost. The NL intervention had a greater impact particularly on reductions in OD through stimulating greater latrine sharing. Both of these outcomes were assessed 1-year after the interventions ended, which does not necessarily indicate longer-term sustainability of the outcomes. This highlights the fact that for interventions that yield multiple outcomes, the results of cost effectiveness analyses are sensitive to the choice of the outcome measure (Boardman et al., 2010). Our study thus reinforces the limitations of using cost effectiveness analyses to guide decision making. Given the importance of high sanitation for health impact, and that sanitation has been recognized as a human right, the most cost effective intervention may not be preferred by policy makers if a less cost effective intervention yields higher coverage. Cost effectiveness analysis can reduce the uncertainty of the consequences of a given decision, but cannot reflect the values of those that will be affected by the decision and the constraints of those making the decision (Wilkinson et al., 2016). Thus, cost effectiveness can serve as a tool but not the sole determinant for decision making.

In addition to variability observed within and across countries, the relative cost effectiveness of the interventions was sensitive to uncertainty about the costs and outcomes of the respective interventions. The Monte Carlo simulations show considerable overlap in the 95% confidence intervals for cost effectiveness of the respective CLTS interventions for both outcome measures. Thus, in addition to considering contextual factors that may influence outcomes, decision makers must be mindful of uncertainty about both costs and the effectiveness of interventions. In situations where costs, outcomes, or both are subject to considerable uncertainty, decision makers should be cautious when using point estimates to guide decision making and consider the distribution of potential outcomes.

### 4.1 Limitations

This study has a number of limitations. Cost effectiveness values are from Ghana and Ethiopia, and may not be generalizable to other contexts. Changes in open defecation and latrine ownership in Ghana are based on recall of latrine age. Outcomes were measured at the household rather than individual level, which does not account for intra-household variation. Households reporting using a latrine observed to be unusable were categorized as OD, which may overestimate OD. Local costs incorporate assumptions about value-of-time. For further details and limitations of the study designs and data collection, refer to prior publications that go into more detail on measuring outcomes and cost (Crocker et al, 2016a, 2016b, 2017b).

### 4.2 Conclusion

There is limited cost effectiveness evidence for sanitation interventions, and the existing evidence generally uses top-down costing methods, and reports aggregate outcomes. We contribute to filling this evidence gap by performing cost effectiveness analysis of CLTS interventions in Ethiopia and Ghana using bottom-up costing, and report results disaggregated by intervention, outcome, and geographical region.

Both the pilot interventions (Teacher CLTS and CLTS + NL Training) involved training more local actors than was conventional in Ethiopia and Ghana. In Ethiopia, the pilot intervention was 30% more cost effective at reducing open defecation. In Ghana, the pilot intervention was 8% less cost effective at reducing open defecation, but had two-to three-times more impact. The NL intervention may be more appealing to policy-makers as reaching high community-level coverage and use of sanitation is both important and challenging (Fuller and Eisenberg, 2016; Garn et al., 2017; Harris et al., 2017). We conclude that the extra cost of building local capacity in the pilot interventions was justified by improved outcomes, and improved effectiveness for the primary outcome targeted by the approach (defecation practices).

Our results demonstrate the extent to which aggregate measures can mask considerable variability in costs and outcomes across countries and regions. We demonstrate the need for economic analyses that attend to local contextual factors and suggest that exploring variability of economic results is more valuable than considering solely aggregate results (Jeuland and Pattanayak, 2012). Instead of reporting just aggregate results, studies should report outcomes for different geographic regions within the study, and should report demographic characteristics of study participants by region as well. Point estimates of economic measures need to be interpreted with care. Both the costs and outcomes of interventions are subject to variability and uncertainty which should be reflected in economic analysis.

Cost effectiveness analysis requires the analyst to choose a single outcome measure. The focus on individual outcomes inhibits comparing multiple potential outcomes that might be considered (Berman, 1982; Briscoe, 1984; Fuente et al., 2012). For CLTS, in addition to the two outcomes we present, this includes handwashing practices, solid waste management, time savings from stopping OD, and improvements in well-being associated with enhanced social status and safety for women. In such instances, cost effectiveness does not provide sufficient guidance to policy makers on how to proceed. While cost effectiveness analysis can and should be used to inform public health policy and programming, it should not be treated as a standalone tool for determining resource allocation.
