# Supplementary material for: Cost effectiveness of community led total sanitation in Ethiopia and Ghana
Source: Int J Hyg Environ Health. 2021 Mar;232:113682. doi: 10.1016/j.ijheh.2020.113682 (PMC7873587; doi:10.1016/j.ijheh.2020.113682)
Supplement: Multimedia component 1 [file mmc1.docx]

**Appendix**

**Authors:** Jonny Crocker, David Fuente, Jamie Bartram

**Manuscript title:** Cost effectiveness of community led total sanitation in Ethiopia and Ghana

**Pages:** 8

**Tables:** 4

**Figures:** 0

**Table A1: Reporting information according to the CHEERS guideline.**

| 1. Title | Cost effectiveness of community led total sanitation in Ethiopia and Ghana |
| --- | --- |
| 1. Abstract | See abstract of main text |
| 1. Background and objectives | See introduction of main text |
| 1. Target population and subgroups | *Selection rationale for Ethiopia:* A pair of regions where Plan had prior CLTS experience and government collaboration were selected: Oromia and the Southern Nations, Nationalities, and Peoples’ (SNNP) regions. One district with no prior CLTS was selected from each region (Deksis and Dara districts, respectively). A total of three road-accessible kebeles with no major towns and low reported latrine access in the 2011 census were selected from each district.  *Selection rationale for Ghana*: The Central, Upper West, and Volta regions in  Ghana were selected for inclusion in this project as they have different environmental and social characteristics, had high levels of OD, and Plan had an established relationship with regional government. One district was selected from each region in which the local government was familiar with CLTS and most villages had not received a CLTS intervention. Twenty villages with no prior CLTS, and a population of 300−1000 according to district records were randomly selected from each district.  *Population characteristics*: Details on target population and subgroup characteristics, and additional details on sample selection, can be found in the main text at the beginning of the results section, in Table 1, and also in prior publications.(Crocker et al., 2016a, 2016b) |
| 1. Setting and location | The setting and location are described in the introduction (CLTS background), and in Table 2. |
| 1. Study perspective | This study adopts a societal perspective for costs. This includes program and local costs. Program costs were those borne by Plan, which included management, training, and facilitation. Local costs were those borne by local actors (district government, teachers, health workers, community members), comprising the economic value of their time, and households’ financial expenditures on latrines. |
| 1. Comparators | Four different CLTS interventions were implemented: in Ethiopia, (1) health extension worker and kebele leader-facilitated CLTS (“HEW CLTS”), and (2) teacher-facilitated CLTS (“Teacher CLTS”); and in Ghana, (3) NGO-facilitated CLTS (“NGO CLTS”), and (4) NGO-facilitated CLTS with additional training for natural leaders (“CLTS + NL Training”). We use “do nothing” as our base case, and present cost efficiency results in absolute terms in the main text. Supplement table 2 presents incremental cost efficiency results. |
| 1. Time horizon | *Ethiopia*: All costs occurred from September 2012 to September 2013, during Plan International Ethiopia’s intervention. Outcomes were assessed from September 2012 to September 2014.  *Ghana*: All costs occurred from October 2012 to May 2014. Outcomes were assessed from October 2012 to May 2015. |
| 1. Discount rate | No discount rate was used as costs were evaluated over a 2 year period (2012 to 2014), and outcomes are not converted to dollar values. |
| 1. Choice of health outcomes | The outcomes used in this study are ownership of a usable private latrine, and latrine use (stopping open defecation). Latrine ownership and use are widely used outcomes in sanitation research, and mirror the targets and monitoring as part of the Millennium Development Goals and Sustainable Development Goals.(WHO/UNICEF, 2017) There are no established methods for converting these outcomes into health outcomes, so we do not convert them. |
| 1. Measurement of effectiveness | This study uses outcome data taken from two prior evaluations, as the objective was to analyze the cost efficiency of the projects as implemented in the geographic settings included in those evaluations.  The evaluation in Ethiopia used a quasi-experimental design, in which kebeles (clusters of villages) were pre-matched on latrine access and population, then manually assigned to receive CLTS facilitated by either HEWs and kebele leaders, or by teachers. The evaluation in Ghana used a cluster-randomized design, in which all project villages received CLTS, and half of the villages were randomly selected to receive natural leader training as an add-on activity. The interventions in Ethiopia took place in the Oromia and Southern Nations, Nationalities, and Peoples (SNNP) regions, and in Ghana in the Central, Upper West, and Volta regions.  In Ethiopia, a complete village listing was conducted at baseline,  then villages were randomly sampled and all households within sampled villages were surveyed. The same households were resurveyed immediately after the interventions and again one year later. In Ghana, a complete household listing was conducted immediately after the interventions, then households were randomly sampled, surveyed for the midline, and resurveyed a year later for the endline. No baseline survey was used in Ghana. Sanitation outcomes were assessed by asking heads- of-households where members of their family primarily defecated and their handwashing practices. Those reporting using a latrine were asked a series of questions to determine if it was private, shared, or communal. Latrine and handwashing station quality and maintenance were then assessed by observation. All data collection was conducted by an independent contractor in each country. |
| 1. Measurement and valuation of preference-based outcomes | We do not measure or value preference-based outcomes. |
| 1. Estimating resources and costs | The cost analysis is reported fully in an already-published article.(Crocker et al., 2017b) |
| 1. Currency, price date, and conversion | Costs occurred in 2012 to 2014, and were not adjusted to a single year. Currency conversions from costs in Ethiopian Birr and Ghanaian Cedis to US dollars was done using the exchange rate from the first day of the month in which the costs occurred. |
| 1. Choice of model | We did not use a decision-analytic model in this study. |
| 1. Assumptions | We did not use a decision-analytic model in this study. |
| 1. Analytical methods | All analytical methods are described in the methods section of the main text. |
| 1. Study parameters | *Inputs:*  All study input parameters and ranges, including further details on value-of-time, are available in the online supplement to the previously published cost analysis, available online at: <http://dx.doi.org/10.1016/j.scitotenv.2017.05.279>.(Crocker et al., 2017b) Value-of-time ranges by country and actor are below:  Value-of-time, Ghana   - Community members and natural leaders: $1.06 - $1.60/day, which is half of the national minimum wage pre-2015. The range is due to varying exchange rates over the course of the project - Hired labor: $0.50-$1 / hour. The low end is used in the Upper West region. Based on reports from Plan International staff. - District government: $3.13 / hour. Based on Plan International compensation rates for district government during training.   Value-of-time, Ethiopia   - Community members and natural leaders: $0.07 / hour, which is half of the public sector minimum wage during the study period. - Health extension workers, teachers, kebele leaders: $0.29 - $0.41 / hour, based on health extension worker wages.   *Outcomes:* Outcome means and ranges are presented in Table 3 in the main text.  *Sensitivity analysis:* Parameters and assumptions used for the sensitivity analysis are presented in a table at the end of the supplement. |
| 1. Incremental costs and outcomes | Presented in Table 3 in the main text. |
| 1. Characterizing uncertainty | Sensitivity of cost-efficiency to uncertainty about the costs and outcomes of the programs was assessed using Monte Carlo analysis, using the assumptions summarized in the supplement. Costs were measured, not estimated, so a uniform distribution with cost range of +/- 30% from base values presented in Table 1 was chosen as a conservative approach for sensitivity analysis. For outcomes, normal distributions using standard deviations taken from impact evaluation data were used. Standard deviations for changes in open defecation in Ghana were not available, so 50% of the point estimates were used for the Monte Carlo analysis. Monte Carlo analysis was implemented using Oracle Crystal Ball and 1000 draws of the parameter values. |
| 1. Characterizing heterogeneity | Variability is assessed by reporting costs, outcomes, and cost efficiency over two different outcomes, and by geographic sub-groups (two regions in Ethiopia, and 3 regions in Ghana). |
| 1. Study findings, limitations, generalizability, and current knowledge | The findings, limitations, generalizability, and comparison to current knowledge are presented in the discussion section in the main text. |
| 1. Source of funding | This research is a result of a sub-agreement to the University of North Carolina at Chapel Hill from Plan International USA, which received a grant from the Bill & Melinda Gates Foundation (OPP1028953). |
| 1. Conflicts of interest | The authors declare no real or potential conflicts of interest. |

This table is adapted from (Crocker et al., 2017a) with permission:

**Table A2. Household and respondent characteristics in villages receiving CLTS in Ethiopia and Ghana, by intervention.**

| **Variable** | **Ethiopia** | |  | **Ghana** | |
| --- | --- | --- | --- | --- | --- |
|  | **CLTS** | **Teacher CLTS** |  | **CLTS** | **CLTS + NL training** |
| Female respondent | 73% | 77% |  | 74% | 69% |
| Five or more years of education^a^ | 20% | 17% |  | 52% | 58% |
| Household size (people) | 6.1 | 5.7 |  | 4.1 | 3.9 |
| Number of children per household | 0.9 | 0.9 |  | 0.7 | 0.6 |
| Metal roof | 28% | 19% |  | 88% | 93% |
| Own radio | 26% | 27% |  | 48% | 50% |
| Own television | 1% | 1% |  | 34% | 41% |
| Years family lived in village | 24 | 21 |  | 31 | 25 |
| Years family lived in current house | 15 | 13 |  | 15 | 14 |
| Use improved water supply | 51% | 51% |  | 77% | 77% |
| Baseline latrine ownership | 84% | 76% |  | 9% | 13% |
| Baseline open defecation | 38% | 48% |  | 49% | 49% |
| Abbreviations: NL, natural leader. ^a^Assumes that respondents who have completed primary education in Ghana have spent at least five years in education. All analysis accounts for unequal selection probabilities, non-response rates, and village-clustering. All Ethiopia values are from the baseline survey. All Ghana values are taken from the midline survey, and describe the two treatment groups at that time, except for latrine ownership private latrine ownership, which is based on recall of how old their latrines were. | | | | | |

**Table A3. Incremental cost-efficiency ratios (ICER) of conventional and pilot CLTS interventions in Ethiopia and Ghana.** The ICER for conventional CLTS is in comparison to "do nothing". The ICER for pilot CLTS is in comparison to conventional CLTS.

|  | Ethiopia | | |  | Ghana | | |
| --- | --- | --- | --- | --- | --- | --- | --- |
| Variable | Do nothing* | HEW CLTS (conventional) | Teacher CLTS (pilot) |  | Do nothing* | NGO CLTS (conventional) | CLTS + NL training (pilot) |
| Cost | $0 | $36,739 | $63,348 |  | $0 | $131,783 | $344,190 |
| Households stopping open defecation | 0 | 168 (10.4%) | 421 (11.0%) |  | 0 | 298 (8.7%) | 718 (21.7%) |
| Households gaining access to a usable latrine | 0 | 70 (4.3%) | 229 (6.0%) |  | 0 | 305 (8.9%) | 481 (14.5%) |
| ICER (open defecation) | Comparator | $218.26 | $105.52 |  | Comparator | $442.26 | $505.23 |
| ICER (latrine ownership) | Comparator | $522.02 | $167.67 |  | Comparator | $432.11 | $1,205.38 |
| Incremental cost efficiency (ICER) here refers to the cost to convert a single household from open defecation pre-intervention (baseline) to latrine use 1-year after the intervention ended (endline), or to the cost for a single household to gain access to a private, usable latrine over the same time period. The cost takes a societal perspective, comprising program costs and locally borne costs. *The "Do nothing" comparator assumes that there would have been no change in open defecation or latrine ownership in the absence of the interventions. | | | | | | | |

**Table A4: Sensitivity analysis parameters.**

|  | **ETHIOPIA** | | | | | | **GHANA** | | | | | | | |
| --- | --- | --- | --- | --- | --- | --- | --- | --- | --- | --- | --- | --- | --- | --- |
|  | Combined | | Oromia | | SNNP | | Combined regions | | Central | | Upper West | | Volta | |
| **Parameter (*distribution*)** | HEW-CLTS | Teacher-CLTS | HEW-CLTS | Teacher-CLTS | HEW-CLTS | Teacher-CLTS | CLTS | CLTS + NL training | NGO CLTS | NGO CLTS + NL training | NGO CLTS | NGO CLTS + NL training | NGO CLTS | NGO CLTS + NL training |
| Cost (uniform distribution used) | $36,739 | $63,345 | $20,573 | $30,235 | $16,166 | $33,110 | $131,773 | $344,169 | $45,797 | $110,747 | $33,801 | $106,452 | $52,175 | $126,970 |
| Min | n.a. | n.a. | $14,401 | $21,165 | $11,316 | $23,177 | n.a. | n.a. | $32,058 | $77,523 | $23,660 | $74,517 | $36,523 | $88,879 |
| Max | n.a. | n.a. | $26,745 | $39,306 | $21,016 | $43,043 | n.a. | n.a. | $59,536 | $143,971 | $43,941 | $138,388 | $67,828 | $165,061 |
| Change in open defecation - mean (normal distribution used) | -10% | -11% | -48% | -56% | 15% | 21% | -9% | -22% | -6% | -16% | -13% | -53% | -9% | -15% |
| Standard deviation* | 3.86% | 4.02% | 5.61% | 3.05% | 4.67% | 6.54% | 4.34% | 13.04% | 3.06% | 7.92% | 6.71% | 26.54% | 4.27% | 7.63% |
| Change in ownership of a usable latrine - mean (normal distribution used) | 4% | 6% | 43% | 53% | -22% | -27% | 9% | 15% | 7% | 4% | 8% | 33% | 12% | 19% |
| Standard deviation | 4.69% | 3.67% | 5.68% | 2.97% | 6.40% | 5.89% | 1.66% | 2.82% | 2.59% | 3.71% | 3.42% | 7.48% | 3.76% | 6.11% |
| *Monte Carlo analysis was implemented using Oracle Crystal Ball and 1000 draws of the parameter values. Cost minimum and maximum values for the uniform distribution are +/-30% from the base case. Standard deviations are taken from the logistic regression results in the previously published impact evaluations. *Standard deviation not available for change in open defecation in Ghana. Approximated by 0.5 the difference of the 95% CI from parameter estimates.* | | | | | | | | | | | | | | |

**Supplement references**

Crocker, J., Abodoo, E., Asamani, D., Domapielle, W., Gyapong, B., Bartram, J., 2016a. Impact evaluation of training natural leaders during a community-led total sanitation intervention: a cluster-randomized field trial in Ghana. Environ. Sci. Technol. 50, 8867–8875. https://doi.org/10.1021/acs.est.6b01557

Crocker, J., Geremew, A., Atalie, F., Yetie, M., Bartram, J., 2016b. Teachers and sanitation promotion: an assessment of community-led total sanitation in Ethiopia. Environ. Sci. Technol. 50, 6517–6525. https://doi.org/10.1021/acs.est.6b01021

Crocker, J., Saywell, D., Bartram, J., 2017a. Sustainability of community-led total sanitation outcomes: evidence from Ethiopia and Ghana. Int. J. Hyg. Environ. Health 220, 551–557. https://doi.org/10.1016/j.ijheh.2017.02.011

Crocker, J., Saywell, D., Shields, K.F., Kolsky, P., Bartram, J., 2017b. The true costs of participatory sanitation: evidence from community-led total sanitation studies in Ghana and Ethiopia. Sci. Total Environ. 601–602, 1075–1083. https://doi.org/10.1016/j.scitotenv.2017.05.279

WHO/UNICEF, 2017. Progress on drinking water, sanitation and hygiene: 2017 update and SDG baselines. Geneva.
